# Thoracoscopic repair of esophagogastric anastomotic fistula using a pedicled pleural flap: a case report and literature review

**DOI:** 10.3389/fonc.2025.1696165

**Published:** 2026-01-12

**Authors:** Yujian Li, Yongjun Deng, Jianbin Zou

**Affiliations:** 1 Department of Thoracic Surgery, The Affiliated Hospital of Yunnan University, Kunming, Yunnan, China; 2 Graduate School, Kunming Medical University, Yunnan, China

**Keywords:** empyema, esophagogastric anastomotic leakage, minimally invasive esophagectomy, pedicle pleural flap, thoracoscopy

## Abstract

**Background:**

Esophagogastric anastomotic leakage is one of the most serious complications following radical esophagectomy for esophageal cancer. Anastomotic leakage leads to prolonged hospitalization, increased medical costs, reduced quality of life, and higher mortality. Thus, early detection and effective treatment of this complication are crucial. Successful treatment of anastomotic fistula hinges on closing the fistula tract.

**Case Summary:**

We treated a patient with locally advanced mid-thoracic esophageal squamous cell carcinoma. After two cycles of neoadjuvant chemotherapy, he underwent minimally invasive thoracoscopic esophagectomy using the McKeown procedure. However, on postoperative day 11, he developed a left cervical esophagogastric anastomotic leakage that extended into the right thoracic cavity, leading to empyema formation. After controlling the acute infection, we innovatively used a parietal pleural pedicled flap, assisted by video-assisted thoracoscopy, to repair the anastomotic fistula. Additionally, we performed thoracoscopic fiberboard decortication for the empyema. Subsequent measures included continuous low-pressure negative pleural suction in the right thoracic cavity to promote pulmonary re-expansion and eliminate residual cavity, as well as continuous irrigation and drainage to maintain a clean postoperative environment in the right thoracic cavity. These comprehensive treatments led to complete healing of the esophagogastric anastomotic fistula, and the patient had an uneventful recovery without any sequelae.

**Conclusion:**

This case demonstrates that pedicled pleural flaps are a viable and practical option for repairing anastomotic fistulas. Our treatment approach offers advantages of simplicity, practicality, and minimal invasiveness, providing a reference for managing esophagogastric anastomotic fistula patients.

## Background

Esophagogastric anastomotic leakage is one of the most serious complications after radical esophagectomy for esophageal cancer. Despite the continuous advancement of surgical techniques for esophageal cancer and the improvement of perioperative management in recent years, the incidence of esophagogastric anastomotic leakage remains high. According to literature reports, the incidence of cervical anastomotic leakage is between 10% and 21.2%, and the incidence of thoracic anastomotic leakage is between 3% and 17%. Although the incidence of thoracic anastomotic leakage is lower than that of cervical anastomotic leakage, patients with thoracic anastomotic leakage have a worse prognosis and higher mortality rate (1, 2). The occurrence of anastomotic leakage is associated with longer hospitalization time, higher medical costs, lower quality of life, and higher 90 day mortality rate (3, 4), highlighting the importance of prevention or early diagnosis and treatment of anastomotic leakage. We admitted a patient with locally advanced esophageal squamous cell carcinoma located in the middle thoracic esophagus. After receiving two cycles of neoadjuvant chemotherapy, he underwent minimally invasive esophagectomy under thoracoscopy using McKeown procedure, but developed a complication of a left cervical esophagogastric anastomotic leakage entering the right chest cavity after the surgery. we creatively used a pedicled parietal pleural flap transfer to repair the fistula of the anastomotic site under thoracoscopy. At the same time, we performed a thoracoscopic fiberboard decortication for empyema, followed by continuous low-pressure negative pleural suction in the right chest cavity to promote pulmonary reexpansion, and continuous flushing and drainage of the right thoracic cavity after surgery. Through the above comprehensive treatment, the patient’s esophagogastric anastomotic fistula was completely healed, and the patient recovered smoothly without leaving any sequelae. To the best of our knowledge, this is the first report to use a pedicled parietal pleural flap transfer to repair the esophagogastric anastomotic fistula under thoracoscopy.

## Case presentation

**Figure 1:**
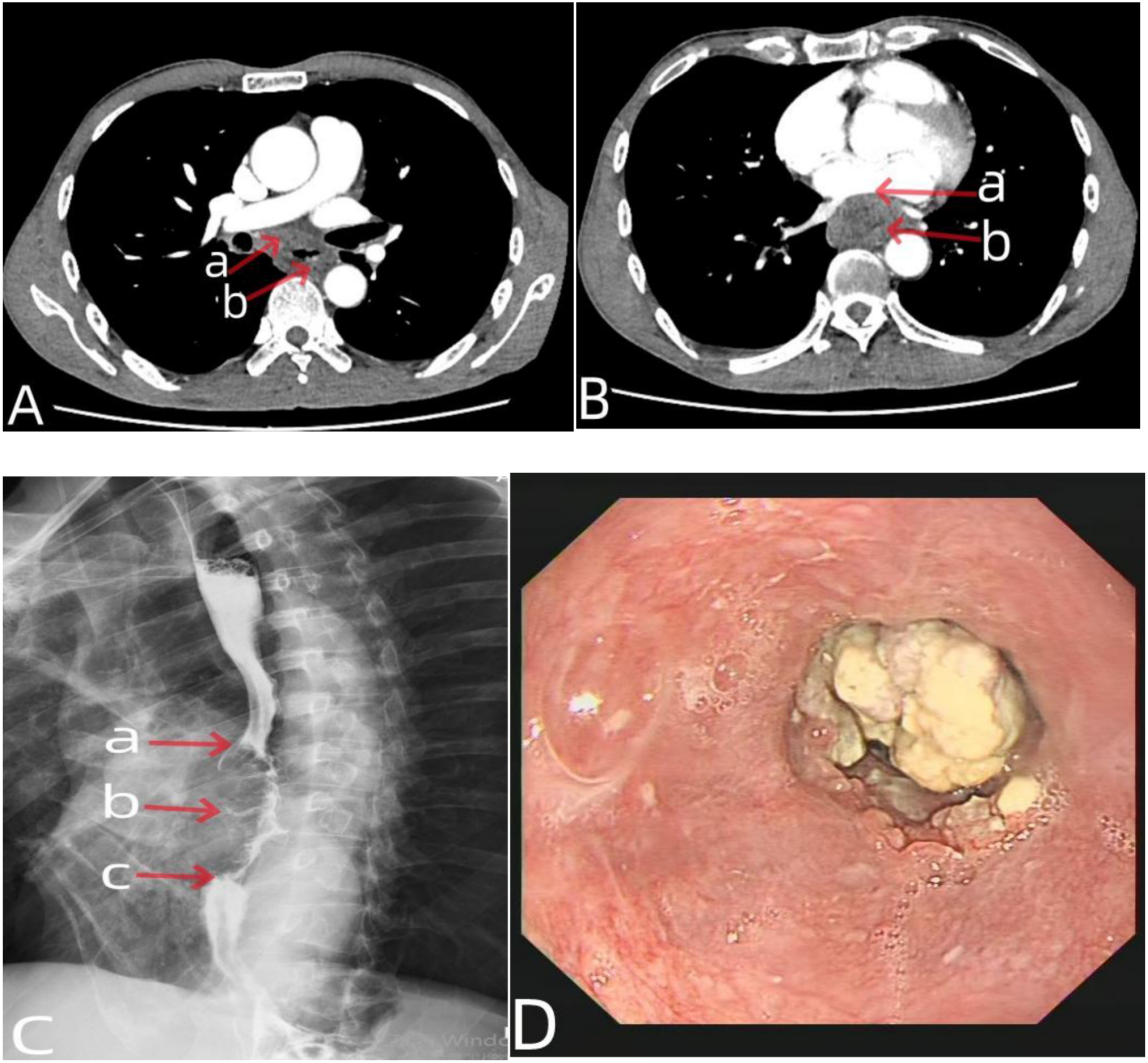
Chest CT, esophagography, and gastroscopy data of the patient before neoadjuvant chemotherapy. In **(A)**, the arrow a represented enlarged subcarinal lymph nodes, the arrow b represented esophageal tumors. In **(B)**, the arrow a represented the left atrium is compressed by tumor, the arrow b represented there was no clear boundary between the tumor and the thoracic aorta. In **(C)**, the arrow a represented the upper edge of esophageal tumor, the arrow c represented the lower edge of esophageal tumor, and the arrow b represented the filling defect formed by esophageal tumors. In **(D)** showed a cauliflower like neoplasm in the esophageal lumen under endoscopy.

**Figure 2:**
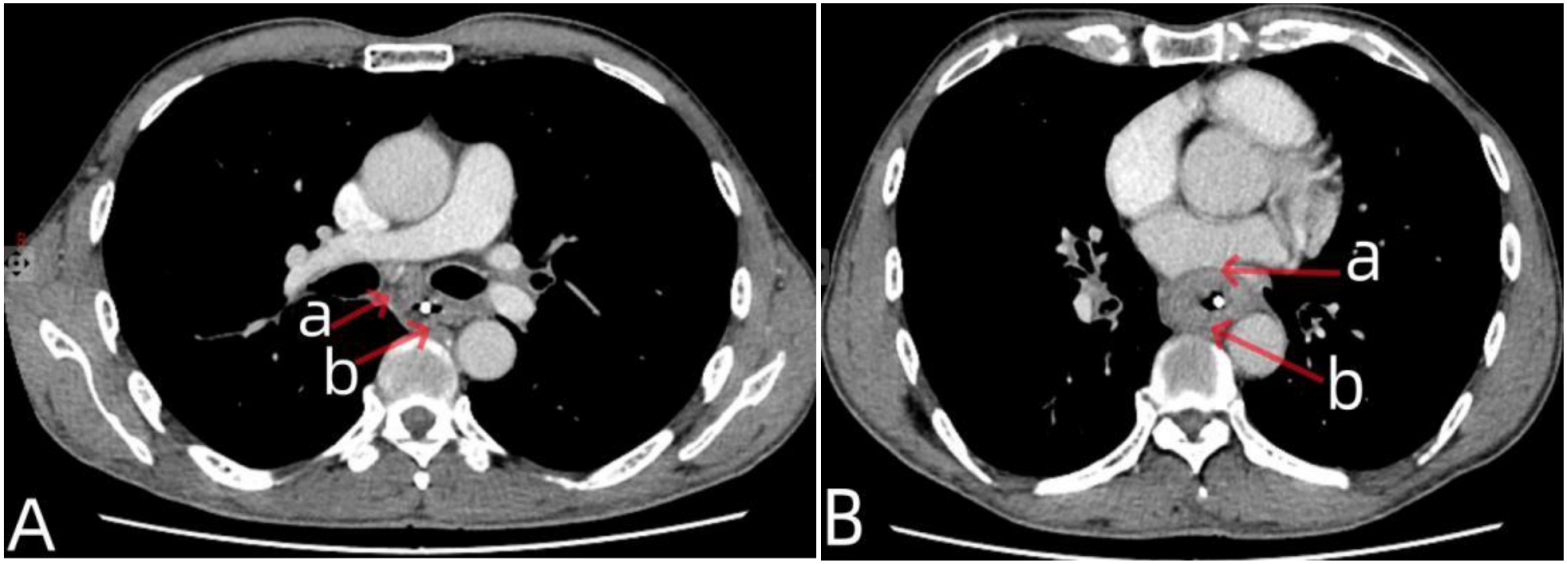
Chest CT data of the patient after neoadjuvant chemotherapy. In **(A)** the arrow a indicated that the enlarged subcarinal lymph nodes had shrunk after chemotherapy, the arrow b represented the esophageal tumor that had shrunk after chemotherapy. In **(B)** the arrow a indicated that there was a clear boundary between the esophageal tumor and pericardium, the arrow b indicated that there was a boundary between the esophageal tumor and the aorta.

A 48 year old male patient was admitted to the hospital due to gradually worsening dysphagia for over 7 months. He had a long-term history of drinking and smoking, and he also had a history of hypertension for more than 5 years. Chest CT scan showed that the esophageal wall thickness at middle thoracic esophagus and subcarinal lymph nodes enlargement. The tumor had infiltrated the entire esophageal wall, and the esophageal lumen was completely blocked. Chest CT also showed that there was no clear boundary between the tumor and the thoracic aorta, and the left atrium is compressed by tumor(**Figured 1A, B**). Esophagogram showed mucosal damage and irregular filling defects in the middle thoracic esophageal lumen (**Figure 1C**). Endoscopy showed a cauliflower like neoplasm completely blocking the esophageal lumen at a distance of 29cm from the incisors. Pathological examination with biopsy revealed moderately differentiated squamous cell carcinoma (**Figure 1D**). We placed a nasogastric tube under endoscopic guidance to provide nutrition to the patient. The preoperative diagnosis was cT3N1M0 stage III middle thoracic esophageal cancer according to the 8th edition UICC staging of esophageal cancer (5). Due to the locally advanced stage of the disease, the patient received two cycles of neoadjuvant chemotherapy with albumin-bound paclitaxel combined with cisplatin regimen. Three weeks later, a chest CT scan was reexamined, and it was found that the esophageal tumor and subcarinal lymph nodes had shrunk, and there were clear boundaries between the esophageal tumor and thoracic aorta, as well as between tumor and pericardium (**Figures 2A, B**). Subsequently, we performed a minimally invasive esophagectomy with the McKeown procedure for the patient. During the operation, a circular stapler was used to anastomose the tubular stomach and esophagus at the left neck. The postoperative pathology confirmed a moderately differentiated esophageal squamous cell carcinoma. The pathological stage was pT3N1M0 (G2), corresponding to stage IIIB according to the AJCC 8th edition. Among 32 lymph nodes dissected, two were involved by metastasis (one located at the right recurrent laryngeal nerve and the other at the subcarinal region).

**Figure 3:**
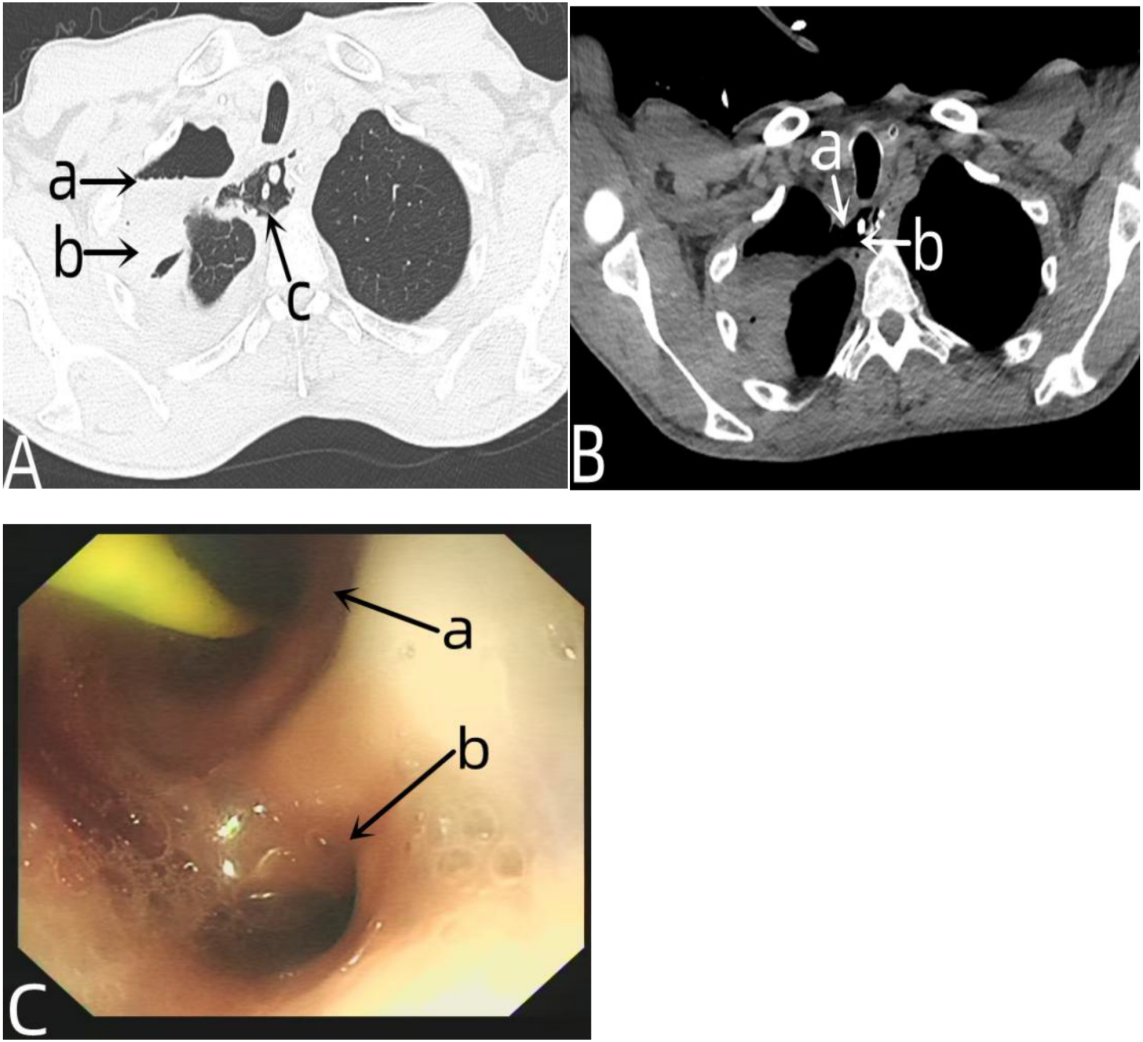
Chest CT and gastroscopy data of the patient with anastomotic leakage. In **(A)** the arrow a indicated the gas-liquid plane in the right thorax, the arrow b indicated the pleural effusion, and the arrow c indicated the tubular stomach in the thorax. In **(B)** the arrow a represented the fistula opening of the anastomotic site, and the arrow b represented the tubular stomach in the thoracic cavity. In **(C)** the arrow a and the arrow b respectively represented the lumen of the tubular stomach and the fistula of the anastomotic leakage observed under gastroscopy.

The patient received nutritional support treatment after the operation, including enteral nutrition via nasojejunal nutrition tube and parenteral nutrition via vein, and was also given antibiotic intravenous injection to prevent infection. The patient suddenly developed high fever and right chest pain on the 11th day after operation, and turbid liquid was drained from the closed drainage tube of the right thorax. The patient’s peripheral blood cell analysis showed that the white blood cell count was 20.09×10^9^/l, the value of procalcitonin was 0.698ng/ml, and the value of C-reactive protein reached 103.8mg/ml. Chest CT revealed a hydropneumothorax and pleural thickening in the right thoracic cavity (**Figure 3A**), and a fistula with a diameter of about 1.5cm was found at the esophagogastric anastomotic stoma (**Figure 3B**). After conducting bacterial culture and drug sensitivity tests on the pus samples, we selected sensitive antibiotics to treat the infection and used physiological saline to flush the right thoracic cavity. The patient’s temperature gradually recovered from high fever to normal. Then the patient underwent gastroscopy, which confirmed that there was a circular fistula with a diameter of about 1.5cm on the right side of the esophagogastric anastomotic stoma (**Figure 3C**).

**Figure 4:**
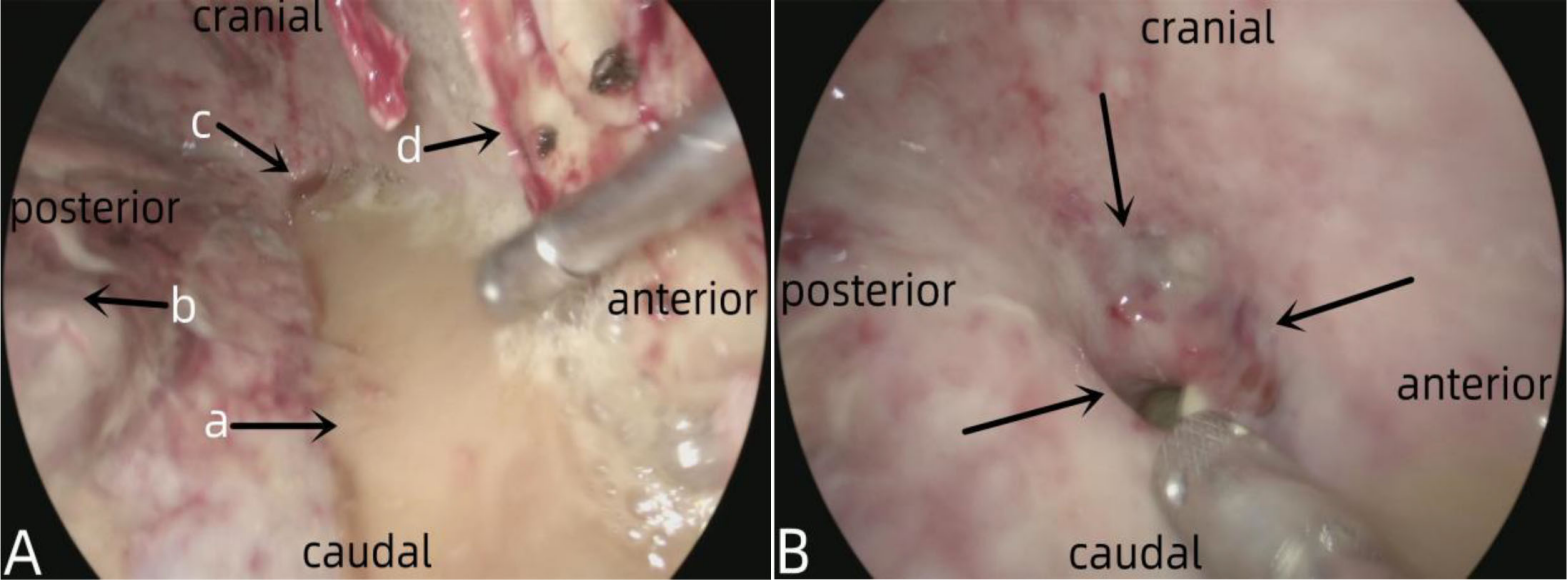
Showed chronic localized empyema caused by anastomotic leakage into the right thoracic cavity. In **(A)** the arrow a indicated the pus, the arrow b denoted the lung tissue, the arrow c represented the fistula, and the arrow d signified the fiberboard of the empyema. In **(B)** the arrows indicated the anastomotic fistula.

**Figure 5:**
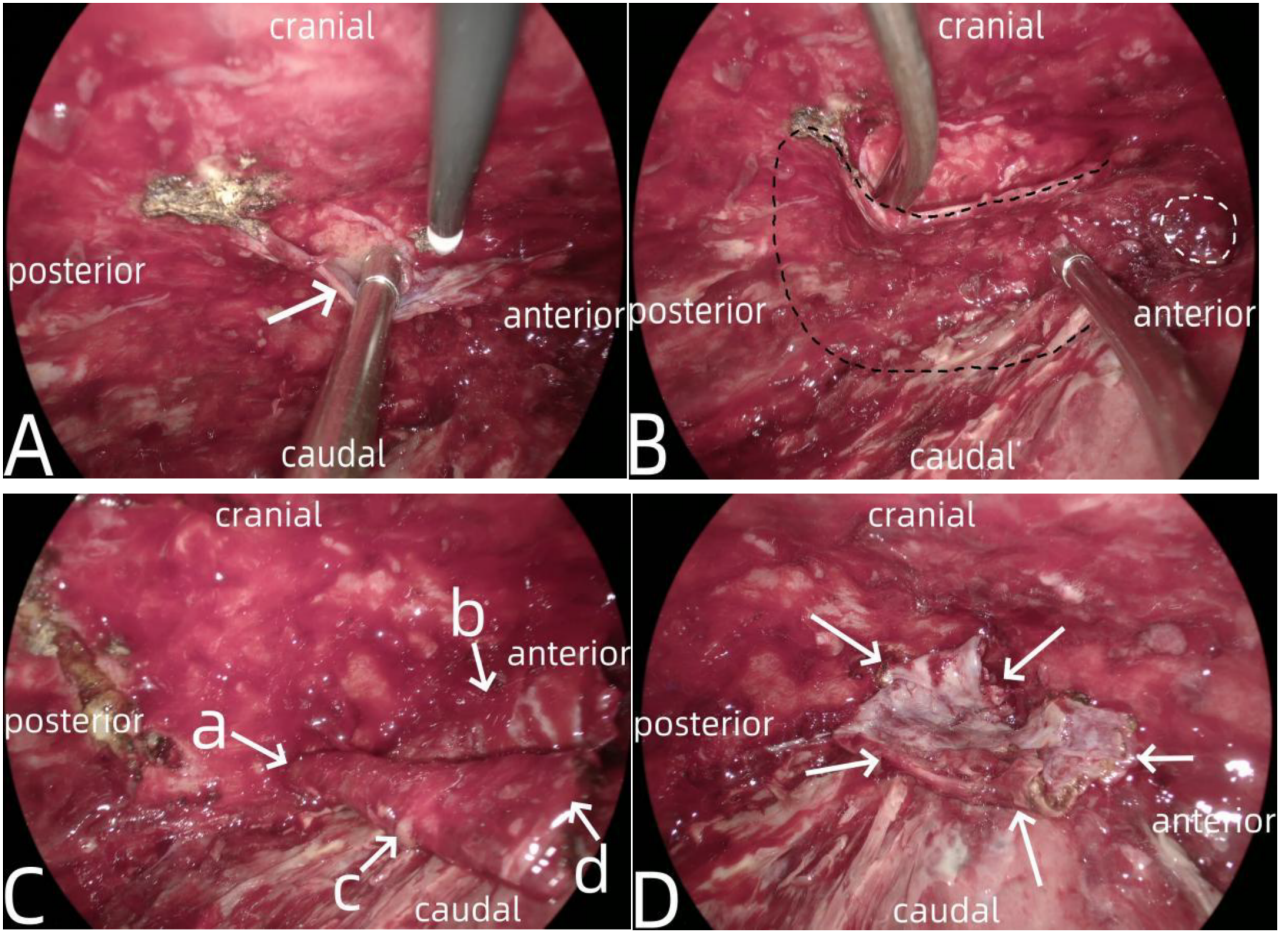
Showed the manufacturing process of pedicled pleural flap. In **(A)** the arrow a indicated the thickened pleura. In **(B)**, the area marked by the black dotted line was the pleura for preparing the pleural flap, while the area marked by the white dotted line is the fistula. **(C)** showed the prepared pedicled pleural flap, where the arrow a indicated the pedicle of the pleural flap, the arrow b indicated the upper margin, the arrow c indicated the lower margin, and the arrow d indicated the anterior margin. **(D)** showed the pedicled pleural flap completely covering the fistula opening, with four arrows indicating the various margins of the pleural flap.

**Figure 6:**
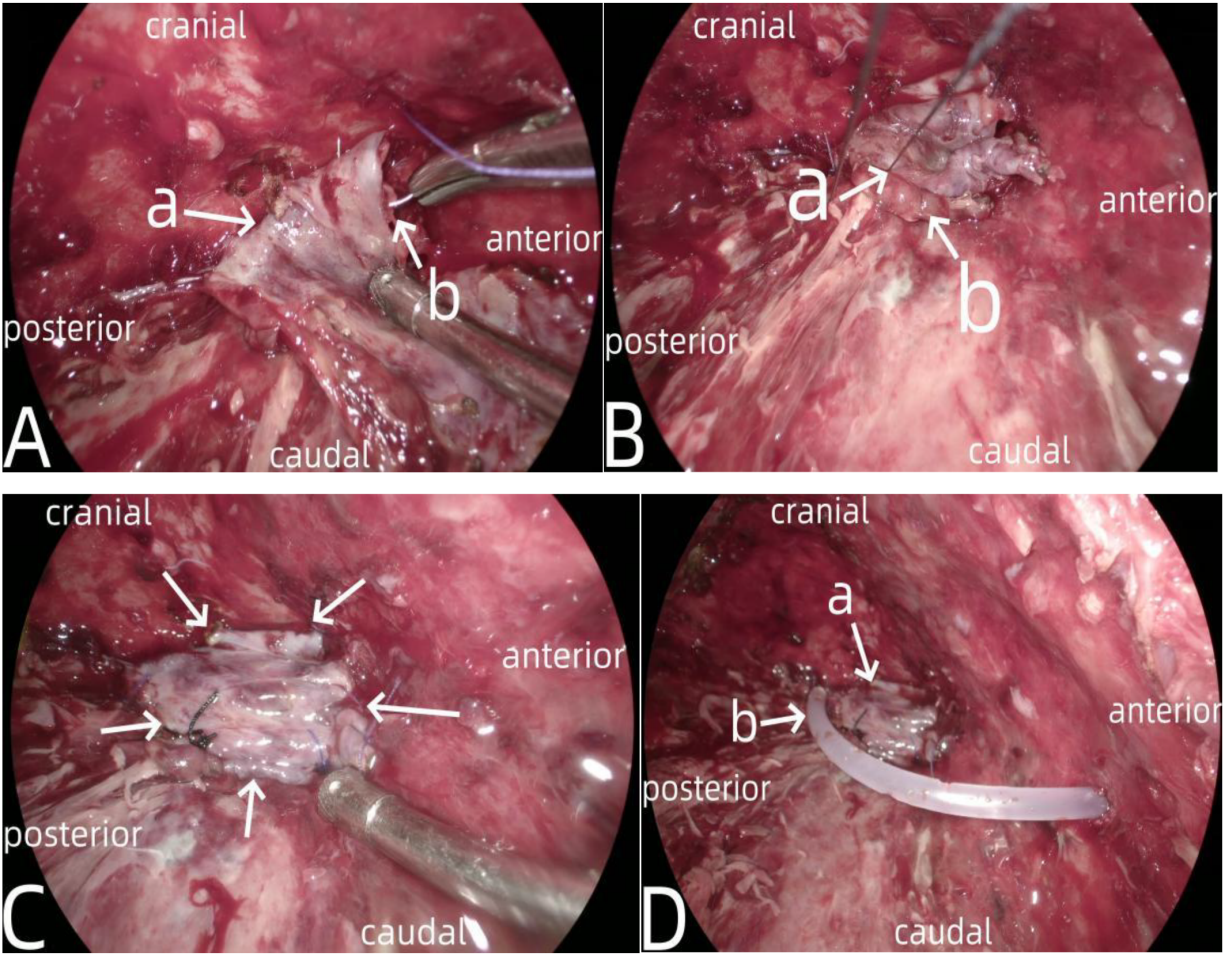
Showed the interrupted suturing and fixation of a pedicled pleural flap around the fistula. In **(A)** the arrow a indicated the pedicle of the pleural flap, and the arrow b indicated the suturing with 3–0 absorbable suture. In **(B)** the arrow a indicated the suturing with 4–0 silk suture, and the arrow b indicated the pleural flap. **(C)** showed the completed suturing, with the arrows indicating the various edges of the pleural flap. **(D)** showed the placement of a drainage tube for thoracic cavity lavage, where the arrow a indicated the pleural flap after suturing and fixation, and the arrow b indicated the 16F drainage tube used as a lavage tube.

Considering that the size of esophagogastric anastomotic fistula was large, and the fistula had leaked into the right thoracic cavity, and the patient was complicated with localized chronic empyema in the right thoracic cavity, we believed that there was little chance of healing the fistula through conservative treatment, so we decided to perform esophagogastric anastomotic fistula repair for the patient. On the 12th day after the occurrence of anastomotic leakage, we performed a single-operation hole thoracoscopic fiberboard decortication for empyema and anastomotic fistula repair with a parietal pleural pedicled flap for the patient. The operation steps were as follows: The patient was placed in a left lateral decubitus position under general anesthesia with dual lumen tracheal intubation. An incision of about 5cm was first made as the main operation port in the third intercostal space between the mid axillary line and the anterior axillary line. The observation hole is located in the sixth intercostal space of the axillary midline. During the operation, we observed a large amount of viscous pus in the abscess cavity of the right empyema (**Figure 4A**). After clearing these pus, it was found that the fistula was located at the top of the right thoracic cavity, with a diameter of about 2cm (**Figure 4B**). We stripped the thickened fiberboard of the parietal and visceral pleurae and cleaned up the necrotic tissue in the right thoracic cavity to prepare for the repair of fistula. However, we found that the tissue around the fistula opening is hard in texture, and the tubular stomach in the thoracic cavity is in a fixed state, making it impossible to directly stitch the fistula opening up. We found that the parietal pleura of the patient was significantly thickened and its blood supply was sufficient (**Figure 5A**), so we decided to use the pedicled parietal pleural flap adjacent to the fistula to repair the fistula. Firstly, we soaked the right thoracic cavity with diluted iodophor solution and repeatedly washed it with a large amount of physiological saline to keep the right thoracic cavity in a relatively sterile state. Then we selected the parietal pleura adjacent to the anastomotic fistula as the target (**Figure 5B**), with the side near the top of the thoracic cavity as the pedicle of the pleural flap, estimated the range of the pleural flap and marked it, and then dissociated the target pleura from chest wall to make a pedicled pleural flap (**Figure 5 C**). Finally, the pedicled pleural flap was covered at the fistula opening (**Figure 5 D**), and sutured to the tissue around the fistula with 3–0 absorbable threads and 4–0 silk threads by interrupted suture method (**Figures 6A–C**). A 24F closed thoracic drainage tube was installed from the observation hole, and a 16F drainage tube was placed at the second intercostal space in the anterior axillary line as a flushing tube, with its end close to the pedicled pleural flap(**Figure 6D**), and the operation was completed. The operation time was about 180 minutes, and the operation was smooth.

**Figure 7:**
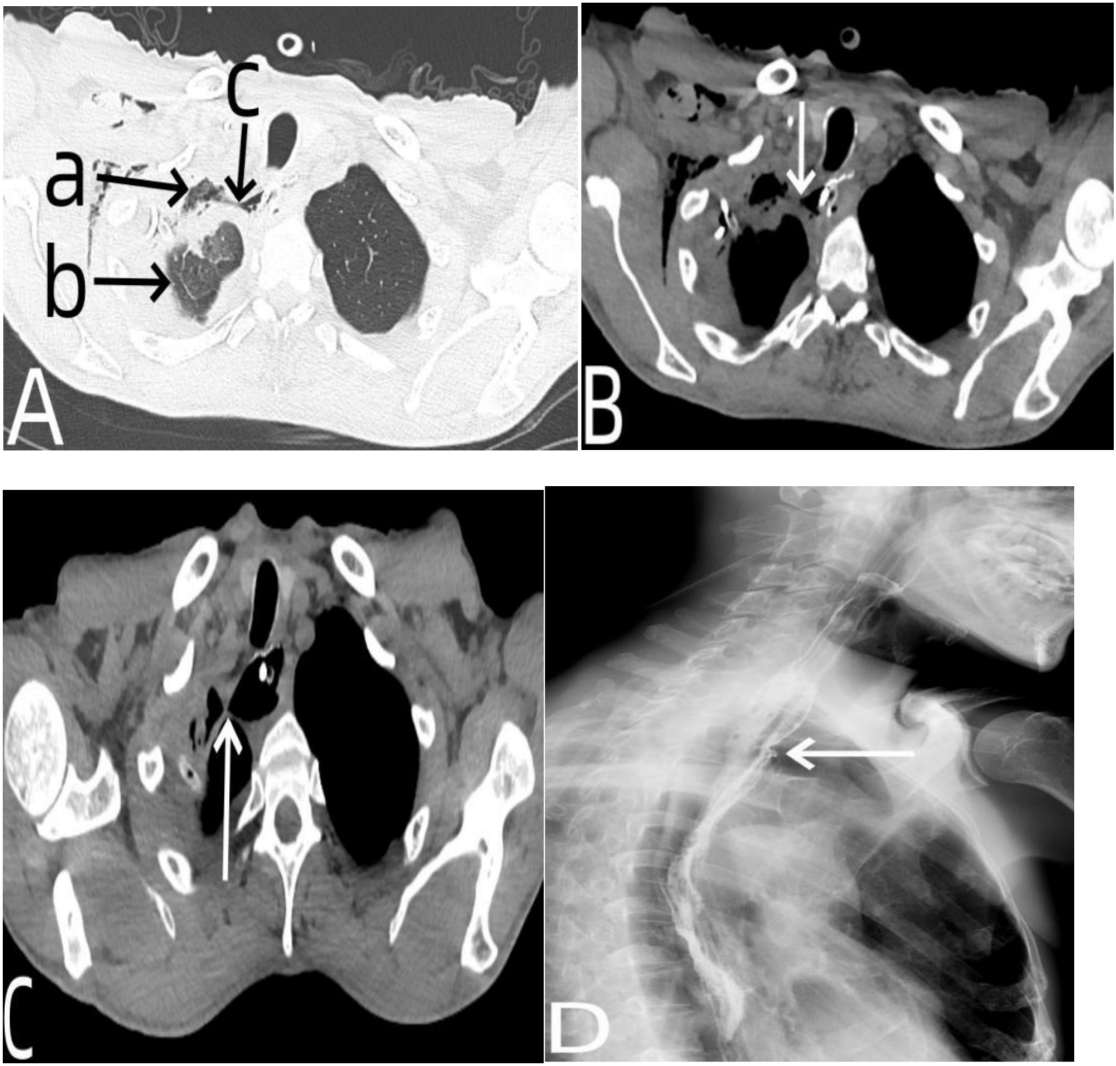
**(A, B)** showed the chest CT scan on postoperative day 9. In **(A)** which was the lung window, the arrow a indicated that the residual cavity in the thoracic cavity had nearly disappeared, the arrow b showed that the right lung had almost fully re-expanded, and the arrow c indicated that the anastomotic fistula had been closed. In **(B)** which was the mediastinal window, the arrow showed that the anastomotic fistula had been closed. **(C)** showed the chest CT on postoperative day 18, with the arrow indicating that the anastomotic fistula had completely closed. **(D)** showed the contrast esophagography on postoperative day 18, with the arrow marking the original site of the anastomotic fistula, where no contrast agent leakage was observed.

After the surgery, the patient received nutrition support treatment including enteral nutrition and parenteral nutrition, as well as anti-infectious therapy based on drug sensitivity testing. In order to keep the right thoracic cavity clean and promote the fistula healing, we dripped 500ml of physiological saline from the flushing tube into the right thoracic cavity to flush the fistula area and repeated this process three times a day. At the same time, in order to promote the rapid reexpansion of lung and eliminate the residual cavity of the empyema, we connected a negative pressure suction device to the right thoracic cavity for continuous low negative pleural suction with a pressure of -1kPa. We observed that the color of the thoracic drainage fluid remained slightly cloudy for the first 6 days after surgery, and gradually became clear from the 7th day after surgery. On postoperative day 9, chest CT showed that the right lung was nearly completely reexpanded, the residual cavity in the right thoracic cavity was significantly smaller, and the anastomotic fistula had been closed (**Figures 7A, B**). On postoperative day 18, the contrast esophagography showed no contrast agent overflowing from the esophagogastric anastomotic stoma, and the chest CT scan indicated that the anastomotic fistula had healed (**Figures 7C, D**). After taking a liquid diet for a day, the patient did not report any discomfort. Subsequently, the closed thoracic drainage tube and flushing tube were removed and the gastric tube was also removed, but the nasojejunal feeding tube was retained. The patient was discharged on postoperative day 21 with the nasojejunal nutrition tube. We required the patient to continue receiving nutritional support therapy by dripping nutrient solution through the nasojejunal feeding tube while taking food orally for 2 weeks after discharge.

**Figure 8:**
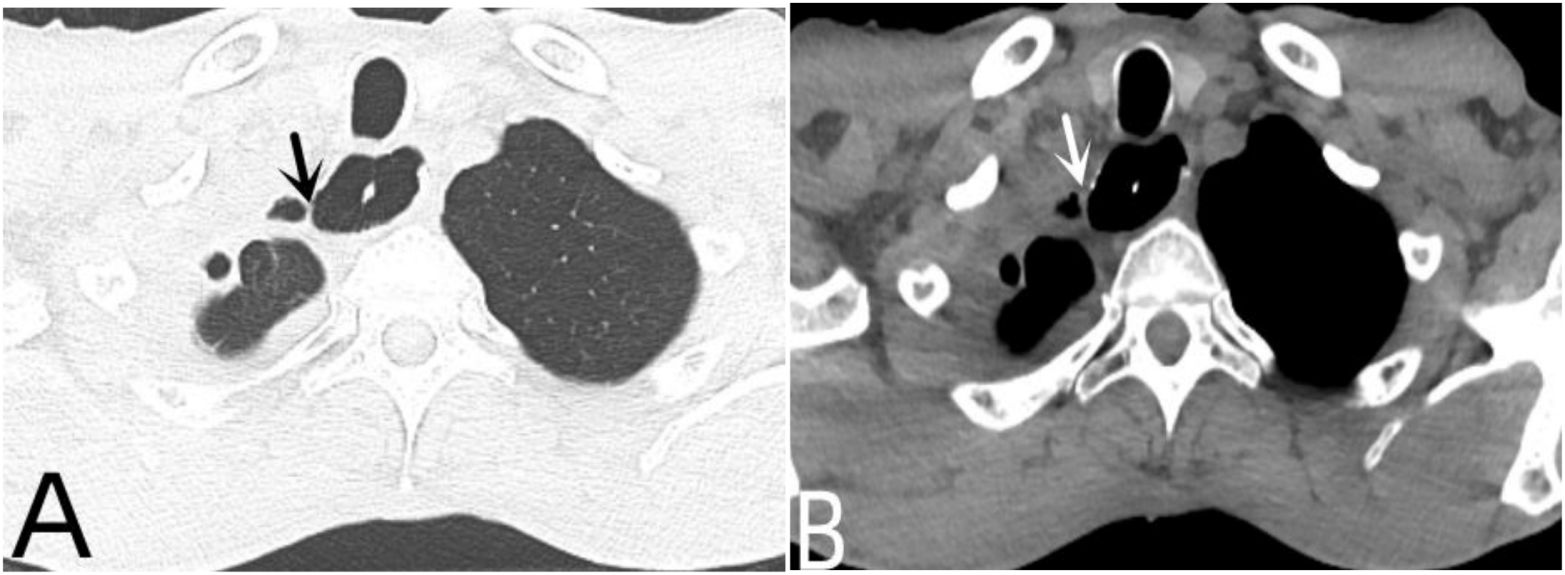
Showed the chest CT scan two weeks after discharge, in **(A)** (lung window) and **(B)** (mediastinal window), the arrows indicated the healed fistula.

Adjuvant therapy and follow-up: The patient underwent a follow-up examination two weeks after discharge, Chest CT scan indicated good healing of the anastomotic stoma and no signs of tumor recurrence (**Figures 8A, B**). Therefore, the nasojejunal feeding tube was removed. Subsequently, the patient received 6 cycles of adjuvant chemoimmunotherapy with albumin bound paclitaxel and cisplatin and Tislelizumab regimen. After the completion of adjuvant chemoimmunotherapy, the patient continued to receive maintenance immunotherapy with Tislelizumab once every 3 weeks for more than 3 months. The patient had a normal diet and no signs of tumor recurrence during a 6-month follow-up period.

## Discussion

Some studies have shown that the occurrence of esophagogastric anastomotic leakage is related to many factors. At present, the risk factors reported mainly included elderly patient, obesity, smoking history, type II diabetes, etc. (6). At the same time, it is also related to factors such as surgical method, the width and blood supply of the tubular stomach, the perioperative serum albumin concentration, the anastomotic site, and whether the anastomotic site is tightly sutured or covered with tissue (such as the greater omentum) (7–10). In addition, neoadjuvant chemotherapy or chemoradiotherapy may have a certain impact on tissue healing, and it is also one of the risk factors for anastomotic leakage (11). Due to the long duration of dysphagia, the patient had complications of malnutrition and hypoproteinemia. Therefore, the risk factors of postoperative esophagogastric anastomotic leakage in this patient included at least smoking history, neoadjuvant chemotherapy and hypoproteinemia.

The pathophysiological changes of cervical anastomotic leakage and intrathoracic anastomotic leakage are not the same. After the occurrence of cervical anastomotic leakage, due to the relatively closed state around the fistula, its inflammatory reaction is limited to the neck soft tissue, which has little impact on the whole body, and the fistula is easy to heal, so the prognosis is good. After the occurrence of intrathoracic anastomotic leakage, the negative pressure of the thorax will suck the liquid of the tubular stomach into the thoracic cavity through the anastomotic fistula, causing pleural infection, empyema, mediastinal infection and sepsis, and the fistula is not easy to heal, so the prognosis is poor and the mortality is high (12, 13). Reviewing the literature, we found that the cases of left neck anastomotic leakage into the right thoracic cavity are not uncommon (14, 15). Since the left neck is connected with the right thoracic cavity during operation, the esophagogastric anastomotic stoma located in the left neck will move down into the right thoracic cavity, so after the occurrence of anastomotic leakage, its pathophysiological changes are similar to those of intrathoracic anastomotic leakage. Therefore, the treatment of anastomotic leakage in this patient is the same as that of intrathoracic anastomotic leakage.

The treatment methods of intrathoracic esophagogastric anastomotic leakage include conservative treatment, endoscopic treatment and surgical treatment. How to choose a treatment plan mainly depends on factors such as the location of the anastomotic fistula, the size of the fistula, the severity of symptoms, and the time of postoperative anastomotic leakage occurrence. A reasonable treatment plan is essential for the prognosis of patients (16). Although in recent years, due to the progress of endoscopic technology, endoscopic self expandable stent implantation plays an increasingly important role in the treatment of esophagogastric anastomotic leakage, the complications of stent detachment or displacement sometimes occur after stent implantation, which cannot play the role of isolating the fistula (17). Surgery is the most effective treatment for repairing fistula, and its key steps include the closure of fistula and the elimination of residual cavity of the thoracic cavity. The methods to close the anastomotic fistula include directly suturing the fistula opening or covering it with tissue flaps. However, due to the edema and infection of the tissue around the fistula, there is little chance of closure the anastomotic fistula by only directly suturing the fistula opening. The pedicled muscle flap has sufficient blood supply and is easy to survive. At the same time, its large volume is conducive to eliminating the residual cavity in the thoracic cavity. Therefore, the success rate of healing the fistula is very high by using the pedicled muscle flap to cover the fistula (18). However, the manufacturing process of pedicled muscle flap is very complex, and the wound is large, which will cause some damage and dysfunction to the patient. In the treatment of the anastomotic leakage of this patient, we did not take the muscle flap as the preferred tissue flap, but creatively used the thickened pedicled pleural flap as the tissue flap to repair the fistula, and successfully closed the fistula, and finally cured the patient. Compared with the pedicled muscle flap, the pedicled pleural flap we used had the advantages of simpler manufacturing process, less damage, more minimally invasive, fewer sequelae, and better quality of life.

In fact, the pleural flap cannot be used as the preferred tissue flap to cover the wound due to some defects. These defects include that the pleura is too thin in thickness, which leads to easy damage when free and sutured, its blood supply is poor, which makes it difficult to survive on the infected wound, and the pleural flap is too small in volume to fill the intrathoracic residual cavity. Reviewing the literature, we found that when tissue flap was needed to cover the fistula in the treatment of esophagogastric anastomotic leakage, the most used tissue flap was actually the pedicled muscle flap, and there were few reports on the use of pleural flap to repair esophagogastric anastomotic fistula (7, 19, 20). However, in the treatment of this patient, we used pedicled pleural flap instead of muscle flap to repair the fistula and achieved success. There were some reasons as follows: 1. the right parietal pleura in this period was in the chronic inflammatory phase, which was significantly thicker than the normal pleura. It was not easy to be damaged when it was made into a pleural flap or sutured, thus maintaining the integrity of the pleural flap. 2.we stitched the pleural flap to the tissue around the fistula tightly to seal the fistula completely, so that the fluid of the intrathoracic tubular stomach would not flow into the thoracic cavity again. 3.In the process of dissociating the pleura, we found that the pleural flap had a good blood supply and is easy to survive. 4.After the fiberboard on the surface of the right lung was peeled off, the compliance of the right lung tissue had been restored. Under the continuous low negative pressure suction to the right thoracic cavity, the lung tissue reexpanded in a short time, so that the residual cavity in the right chest was eliminated. At the same time, the reexpanded right lung tissue compressed the pleural flap, which plays a supporting role on the pleural flap, so that the pleural flap stuck tightly to the fistula, thus promoting the healing of the fistula. In addition, we used physiological saline to continuously flush the right thoracic cavity every day after the operation, which kept the right thoracic cavity in a relatively sterile state. The clean environment played a very important role in the healing of the fistula.

In conclusion, the case presented here highlights the efficacy of the pedicled pleural flap technique in the management of esophagogastric anastomotic fistula. The treatment methods for this patient had a certain degree of innovation. It is not only simple but also minimally invasive, with short recovery time and low cost, without causing severe complications to the patient. The patients have a good quality of life and a favorable prognosis. This case report adds to the growing body of evidence supporting the use of pedicled pleural flaps as a viable option for the repair of anastomotic fistulas in esophageal surgery.

## Data Availability

The original contributions presented in the study are included in the article/supplementary material. Further inquiries can be directed to the corresponding author.
